# *Ex-Vivo* Heart Perfusion Machines in DCD Heart Transplantation Model: The State of Art

**DOI:** 10.3389/ti.2025.12987

**Published:** 2025-08-13

**Authors:** Chiara Tessari, Giovanni Lucertini, Mariangela Addonizio, Veronica Geatti, Daniela Bacich, Nicola Pradegan, Assunta Fabozzo, Roberto Bianco, Giuseppe Toscano, Vincenzo Tarzia, Gino Gerosa

**Affiliations:** Cardiac Surgery Unit, Cardio-Thoraco-Vascular and Public Health Department, Padova University Hospital, University of Padua, Padova, Italy

**Keywords:** heart transplantation, donation after cardiac death, ischemia and reperfusion injury, *ex vivo* heart perfusion, *ex vivo* heart preservation

## Abstract

The Donation-after-Circulatory-Death (DCD) heart transplantation program increases donor pool but resulting in more serious ischemic-related myocardial injury (IRI), leading to higher incidence of primary graft dysfunction (PGD). *Ex-vivo* machine perfusion (EVMP) for DCD heart is being considered a useful aid in improving grafts number and quality assessment, aiming to better outcomes. In this review we will analyze the role of EVMP techniques in the context of DCD with special attention to their clinical aims and results and future perspectives. A review of available clinical and pre-clinical studies involving EVMP with DCD donation model was performed. Thirty-four original articles about preclinical studies were found. First studies were designed to evaluate graft function in DCD hearts after EVMP, while recent research focus on possible therapies that could be associated with EVMP. Twenty-one original articles about clinical studies were found with the Organ-Care-System (TransMedics) as MP used. Outcomes, such as survival rates or rejection episodes, are comparable to outcomes from donation-after-brain-death. EVMP in the setting of DCD heart transplantation can be a valid tool for organ preservation and transport. The role of pre-clinical research will be crucial to reduce IRI, achieve organ reconditioning and reduce incidence of PGD.

## Background

According to most recent worldwide guidelines, heart transplantation (HT) remains the gold standard for treatment of patients with end-stage heart failure (ESHF) [1, 2]. Nevertheless, the discrepancy between organ availability and request is responsible for an unacceptable high awaiting risk mortality [3]. According to the OPTN/SRTR 2021 Annual Data Report, pretransplant mortality rate in US is still 8.6 deaths per 100 patient-years [4]. HT candidates are becoming significantly older, with high levels of morbidity and thus being often bridged to transplantation with mechanical circulatory support [5, 6]. In addition, donor characteristics are changing towards older donors with a worse risk factors profile, resulting in a higher incidence of poorer organ quality, especially in European countries [7]. This increases the need of accepting marginal organs for marginal candidates [8].

Multiple strategies aimed to increase donor pool and improve procured graft quality are being explored, as shown in Table 1. In addition to increasing the absolute number of viable organs, the main etiopathological target of these techniques and technologies is to reduce total ischemia time, the burden of ischemia-reperfusion injury (IRI) and primary graft dysfunction (PGD) [9, 10]. In particular, technologies called *ex-situ* or *ex-vivo* heart preservation (EVHP) technologies, allow the preservation of donor hearts outside the body, and can be used to assess the donor organ as well as to reduce total ischemia time.

**TABLE 1:** Strategies for increasing donor pool and improving procured graft quality.

Donor selection	Graft management
Marginal donors	Graft preservation
Marginal hearts	Pre-transplant graft evaluation
Optimization of allocation policies	Pre-transplant graft “therapy”
Long distance procurement	
DCD (Donation after circulatory death)	

On the other hand, it has been estimated that the use of Donation after Circulatory Death (DCD) hearts could increase the donor pool by approximately 30%–48% [10–13]. The DCD heart transplantation program is already a reality: it consists in using grafts from patients who do not fulfill brain death criteria but have no hope of recovery. In these situations, organ procurement occurs after circulatory arrest. This creates an inevitable phase of warm ischemia [absent in donation after brain death (DBD)] resulting in more serious myocardial IRI, thereby leading to higher incidence of PGD [14–17]. The lack of evidence pertaining best practices in different steps of DCD heart procurement makes it an evolving field. As a matter of fact, in 2020 DBD still represented 97% of donor organs used in the US [4]. *Ex-situ* machine perfusion (MP) for DCD heart is being considered a useful aid in improving grafts number and quality assessment, aiming to better patients’ outcomes. Optimal strategies for cardiac graft assessment are still under evaluation [18]. In this review we will analyze the role of EVHP techniques in the context of DCD with special attention to their clinical aims and results and future perspectives.

## Methods

A review of available studies involving EVHP in human and animal procured with DCD donation model was performed. PubMed was searched to select studies related to this topic. The search terms were: (Transpl*[TIAB] AND (*Ex-vivo* [TIAB] OR *Ex-situ* [TIAB] OR Xvivo [TIAB] OR OCS [TIAB] OR Organ Care System [TIAB] OR perfus* [TIAB] OR preserv*[TIAB]) AND (myocard*[TIAB] OR cardia*[TIAB] OR Heart [TIAB]) AND (DCD [TIAB] OR circula* [TIAB])). The last search was performed up to January 2024. Two authors independently assessed the qualification of the references. A third review author resolved any disagreements between the 2 review authors when necessary. The full text of the retrieved articles and their references were assessed to identify if the studies were adequate to the objective of this review. Statement of Institutional Review Board and Ethics board approval, and Statement of Human and Animal Rights were also checked before inclusion of the study.

A relevant study in this review met the following inclusion criteria: an original study on any kind of DCD with unique data, conducted with EVHP of the grafts in both clinical or preclinical settings, full text written in English only and reported details on the system used and on outcomes. If overlapping data were presented in different publications from the same data source, only the most detailed and relevant articles were included. Abstracts from meetings and other forms of publication were excluded.

### Pre-Clinical Studies Results

Supplementary Table reports the results of the review on original pre-clinical studies involving EVHP in the context of DCD. A total of 34 original articles were identified from 2013 to 2023, involving 774 DCD hearts perfused with MP after procurement. The overall study objectives can be classified into three categories.

• Primarily graft evaluation
• Primarily protocol optimization
• Primarily therapeutic approaches for reconditioning.

Historically, early studies aimed to evaluate graft function in DCD pre-transplant settings. The main objectives of these studies were to assess cardiac viability and performance following a period of warm ischemia, as well as to identify valid indicators of graft injury/quality. Through organ reperfusion, the goal was to identify criteria that could distinguish grafts amenable to heart transplantation from those that were not. The first reported study, dating back to 1997, utilized a custom-made MP device with an erythrocyte-enriched perfusate solution to investigate the effects of normothermic ischemic insults of varying durations [19]. In this study, coronary flow response during reperfusion was considered a key parameter for assessing recovery of power output. Subsequent studies identified additional parameters reflecting organ damage and predictive of myocardial function quality, such as direct hemodynamic or echocardiographic parameters, in particular systolic function, diastolic function, left-ventricular end systolic pressure-volume relationship, left ventricular maximal developed pressure, preload recruitable stroke volume, isovolumic relaxation constant [20–45], histological markers [23, 24, 26, 27, 29, 31, 32, 38–41, 45–47], vascular and microvascular analysis [25–27, 32, 48, 49], laboratory markers indicating metabolism, oxidative stress, etc. of whom the most and easiest used was lactate level [21–24, 30–33, 38–43, 45–48, 50–55] and gene and protein expression [26, 43, 44, 55]. These early studies laid the groundwork for the second category of studies, which focused on the optimization of the primary graft preservation protocol. These studies aimed to identify the most effective organ preservation protocol, including optimal preservation temperature, cardioplegic solution type, and enrichment of the solution with erythrocytes, albumin, etc. Additionally, these studies sought to identify organ evaluation metrics that could predict organ quality for transplant use. Various parameters were identified, including laboratory indices of myocardial damage, oxidative stress, histological markers such as tissue edema and electron microscopy alterations, and the maintenance of adequate echocardiographic or hemodynamic parameters.

Progressive advances in this field shifted research focus from graft evaluation to potential therapies that could be associated with MP to preserve myocardial function and oxygen consumption, reduce ischemia-reperfusion injury (IRI) mechanisms, and facilitate organ reconditioning. Among the therapeutic approaches explored were the use of CytoSorb filters [43], IL-11 [45], methylprednisolone [40], Intralipid [50], autologous mitochondrial transplantation [41], melatonin [42], the NLRP3 inflammasome inhibitor Mcc950 [55], and HSP90 inhibitors [44]. In the early studies, the rat model was predominantly used, whereas later, more advanced translational studies employed porcine models.

### Human Studies Results

Table 2 presents the results of the review on original human studies involving EVHP in the context of DCD. We identified 21 original articles from 2013 to 2023, involving 866 DCD hearts perfused with MP after procurement and subsequently transplanted. A total of 268 DCD hearts were declined for transplantation. All studies used the OCS Heart as the sole MP device in controlled DCD heart transplantation settings. Initially, the purpose of using MP was to reduce ischemia time in distantly-procured transplantations or in recipients with adverse risk factors, such as those on ventricular assist device support [13, 59, 61, 65].

**TABLE 2:** Comparison of perfusion strategies and clinical outcomes in human studies using EVMP in DCD.

Study Design	Study Groups	Perfusion Time (min)	Total Preservation Time (min) total time out of body	Perfusate used Cardioplegia used	Myocardial functional assessment	Outcomes	Article highlights	Reference
Case series	3 DCD	254 min	352 min	Standard OCS perfusate	Lactates trend	30 days survival: 100% (n = 3) moderate cellular rejection: 66.6% (n = 2) Post-operative ECMO: 66.6% (n = 2)	*First successful clinical DCD heart transplantations with donor organs procured at a distance necessitating reanimation, resuscitation, and transportation with use of an ex-vivo cardiac perfusion device*	[56]
Retrospective single-center	13 DCD, 9 transplanted (all donor hearts were transported on the OCS, except for a single heart, where, uniquely, the donor and the recipient were within the same hospital)	205 min	functional WIT of 17.3 min (range, 11–21 min) before cardioplegic infusion. After cardioplegia, an additional 23–28 min of ischemia was encountered while explanting and instrumenting the heart on the OCS before blood reperfusion	Standard OCS perfusate St Thomas’ cardioplegic solution 4 C	Lactates trend	100% survival rate 2/9 requiring MCS (22.2%) No episodes of rejection		[57]
	20 DBD							
Case series	2 DCD OCS with long-term LVAD support	patient 1 360 min	cold ischemic time 13 min + WIT 13 min	Standard OCS perfusate 1L Custodial cardioplegic solution (4C) + 10.000 UI heparin +2500 UI of erythropoietin +50 mg glyceryl trinitrate	Lactates trend	30-day survival: 100% (n = 2) PGD incidence 0% Post-operative ECMO: 0%	*Successful DCD short-term outcomes, despite adverse donor and recipient risk factors, including bridge to transplantation with an implantable LVAD.*	[58]
		patient 2,307	cold ischemic time 12 min + WIT 21 min					
Retrospective single-center	DCD OCS (n = 33/45 DCD heart retrievals), 12 hearts did not progress to circulatory arrest within 30 min from WLS >33 Hearts Retrieved >23 Hearts transplanted +10 Hearts not transplanted (n = 2 OCS failure, n = 8 inadequate recovery)	276 ± 67 min	81 ± 33 min (cross clamp time)	Standard OCS perfusate Cardioplegia St. Thomas’ solution + glyceryl trinitrate (100 mg/L) + erythropoietin (5,000 U/l)	Lactates trend, subjective evaluation of ventricular contractility	Overall survival was 95% at 1 month (n = 22) MCS 39,1% (n = 9) PrimPGD 4.3% (n = 1) Antibody-mediated rejection within the first12 months of transplant was DCD 0.2 ± 0.7 vs. DBD 0.1 ± 0.4 episodes (p > 0.1); acute cellular rejection (ISHLT 2R), DCD 0.8 ± 1.6 vs. DBD 1.0 ± 1.6 episodes (p > 0.1) A total of 7 patients had early acute kidney injury (30%), 4 of whom required short-term hemodialysis (17%)	*Despite a higher requirement for MCS for delayed graft function, primarily in recipients with ventricular assist device support, overall survival and rejection episodes are comparable to outcomes from contemporary brain-dead donors*	[59]
Retrospective single-center	50 DCD heart offers >39 were declined, 3 donors did not proceed to cardiac arrest within the protocol specified time period 8 DCD hearts were retrieved with the OCS, 7 successfully transplanted	median 263 min IQR (242–296)	34 min (IQR 31–39 min]. WIT	Standard OCS perfusate Leucocyte depleting filter Cardioplegia: 500 mL St Thomas crystalloid cardioplegia +50 mg glyceryl trinitrate +2,500 units of erythropoietin	Lactates trend	30-day survival 100% 90-day survival rate was 86% Postoperative MCS with an intra-aortic balloon pump was required in 2/7 (29%) cases and ECMO was required in 3/7 (43%)		[60]
Retrospectivematched, observational cohort study single-center	A. 128 potential DCD donors 78% (100) suffered from cardiac arrest within 4 h −75/100 underwent OCS (18/75 declined) −25/100 underwent TA-NRP (3/25 declined; 19/25 OCS; 3/25 CSS) −79/100 DCD heart transplantation	NRP median 181 IQR (153–200)	warm ischemic time NRP 23 min (21–28)	Standard OCS perfusate Cardioplegia: 500 mL of St. Thomas’s solution +2,500 IU of erythropoietin +50 mg of glyceryl trinitrate	Lactates trend	DCD vs. DBD no difference in 30 days survival (97% DCD vs. 99% DBD, p = 1.00) or 1 year (91% DCD vs. 89% for, p = 0.72) no difference in post-operative ECMO, (15% DCD and 6% DBD for PGD (p = 0.12) no difference in number of treated rejection episodes within the first year after transplantation DPP vs. TA-NRP All recipients in the TA-NRP group remained alive for over the 5 years of the program (survival 100%). For recipients in the DPP group, 30-day survival was 96% (p = 1.00), and 1-year survival was 86% (p = 0.15). Survival was comparable between the 2 retrieval techniques Similar rates of any postoperative MCS (37% vs. 26%, p = 0.58). 18% of DPP and only 5% TA-NRP heart recipients required ECMO support for PGD after HTx (p = 0.27)	*During OCS Langendorff coronary perfusion system allows the heart to beat but not eject and is therefore incapable of a functional assessment*	[12]
		DPP median 251 IQR (221–294)	DPP 36 min (32–43)					
	B. 164 DBD heart transplantations were undertaken, with 50% of heart transplantations performed; CSS preservation			cold crystalloid cardioplegia				
Retrospective single-center	DCD OCS (n = 49/69 DCD heart retrievals); 17 (35%) were declined >32 of the retrieved hearts (62%) were finally accepted for transplantation	281 ± 68 (no ECMO)	cold ischaemic time (29 ± 5) + (281 ± 68) + warm ischaemic time (24 ± 6)	Standard OCS perfusate cold crystalloid St Thomas’ cardioplegic solu- tion containing 100 mg/L of glyceryl trinitrate and 5,000 Units/ L of erythropoietin	Lactates trend	At 5 years of follow-up, the 1-, 3-, and 5-year survival was 96%, 94%, and 94% for DCD hearts compared with 89%, 83%, and 82% respectively for DBD hearts Immediate post-implant requirement for ECMO support for delayed graft function 31% (n = 10); no difference in rejection rates when compared with standard criteria DBD hearts	*Higher lactate values (>5 mM) were acceptable as long as there was a down-trending lactate level* *with an arteriovenous differential in favor of lactate metabolism as an energy source rather than its accumulation indicating signs of myocardial injury*	[61]
		306 ± 60 (ECMO)	Cold ischaemic time (27 ± 6) + (306 ± 60) + warm ischaemic time (23 ± 3)					
Case series	Patient 1 DCD	276	46 (total cross clamp time)	Not specified	*Ex-vivo* coronary angiography	Not specified	*First reports of successful transplantation of DCD allografts following* *ex vivo coronary angiography for the exclusion of significant CAD. External assessment of the coronary arteries alone may have resulted in the exclusion of otherwise viable organs for transplantation. This presents itself as a potentially organ-sparing procedure*	[62]
	Patient 2 DCD	241	69 (total cross clamp time)					
Retrospective single-center	6 DCD + OCS	280	warm ischemic time was 28.5 min	1,500 mL of donor blood + heparin 500 mL of cold crystalloid cardioplegia	Lactates trend, subjective evaluation of ventricular contractility	1-year survival 100% Significant rejection during in-hospital-stay: DCD 2 (33.3%), DBD. 0 (0.0%) (p = 0.143) Need for renal replacement therapy: DCD 1 (16.7%)*, D*BD 2 (22.2%) (p = 1.00) Discharge LV systolic dysfunction grade (mild): DCD 1 (16.7%), DBD 3 (33.3%) (p = 0.6) Discharge RV systolic function grade worse than mild: DCD 1 (16.7%), DBD 1 (11.1%) (p = 1)	*DCD heart transplant can be performed safely with excel-* *lent short-term survival in children; no significant difference in major outcomes compared to a DBD cohort*	[63]
	9 DBD + CSS							
Retrospective single-center	1237 DCD procurements met inclusion criteria (normothermic MP 109, CSS 1128)	Not specified	Not specified	standard OCS perfusate	Not specified	no difference between groups in median total WIT (24.0 min vs. 24.0 min, p = 0.89), but the MPH group demonstrated shorter median operative WIT (circulatory arrest to cross-clamp; 8.7 min vs.10.9 min, p = 0.003) Total actual hearts yield 79; total expected mean yield 3.86; ratio (95%IC): 20.45 (20.45-20.45); p = 0.000 Overall organ yield of DCD heart donors was observed to be 33% higher than expected (O:E 1.33; 95% CI: 1.22–1.45)	*MPH use in DCD procurements does not lead to delays in WIT and does* *not negatively affect organ yield of other concurrently procured organs*	[64]
Retrospective single-center	31 DCD OCS		Cold ischemic time DCD-OCS 94.5 ± 11.0 vs. DBD-OCS 89.2 ± 13.6 vs. DBD-CSS 127.3 ± 63.6; p < 0.050	Standard OCS perfusate	Not specified	6-month survival DCD-OCS 96.8% vs. DBD-OCS 100% vs. DBD-CSS 94.7%; p = 0.637 Post-op MCS:51.6% DCD vs. 31.3% DBD OCD vs. 15.8% DBD CSS; p < 0.050 PGD: 19.5% DCD-OCS vs. 0% DBD-OCS vs. 5.3% DBD-CSS; p < 0.050 6-month Significant rejection >2R: DCD-OCS 25.8% vs. DBD-OCS 18.8% vs. DBD-CSS 34.2%; p = 0.489 Dialysis at time of discharge: DCD-OCS 6.5% vs. DBD-OCS 0% vs. DBD-CSS2.6%; p = 0.493	*There were no differences in cardiac MRI findings between the three transplant types, including presence of gadolinium hyperenhancement after transplant (all p > 0.050)*	[65]
	16 DBD OCS							
	38 DBD cold storage							
Case report	1 DCD	423	WIT 24 min	1.2 L of donor blood been passed through a leucocyte filter with 500 mL of TransMedics Priming Solution containing buffered electrolytes + mannitol, multi-vitamins, antibiotics, albumin, steroids amino acids, dextrose-insulin, and low-dose adenosine 1L of cold del Nido cardioplegia	Lactates trend	Not specified	*Expanding the donor acceptance criteria to include more distant donor locations and enrolling* *recipients with extended criteria (e.g., heart retransplantation) is feasible*	[66]
Retrospective multicenter	157 DCD wit TA-NRP	Not specified	14.9 ± 7.6 (SD)	Not specified	Lactates trend	Survival similar in the taNRP group when compared to DBD. 30-day survival 96.8% ([92.5%–98.6%] 95% CI, n = 156), 1-year survival 93.2% ([87.7%–96.3%] 95% CI, n = 72) and 5-year survival 84.3% ([69.6%–92.2%] 95% CI, n = 13) 12.8% DCD patients required postoperative MCS after transplantation 12.7% for DBD group (p = 1) 7% experienced acute rejection warranting treatment The association between using either CSS or MP in patients receiving taNRP and survival did not differ significantly between groups (HR = 0.33 [0.06; 1.76], p = 0.196) The use of MCS early after surgery differed significantly between the two groups (p = 0.0311). 10.3% CSS required MCS (n = 14.0), vs. 28.6% in MP (n = 6)	*taNRP offers an effective method of organ preservation and procurement*	[67]
	673 DBD							
Retrospective multicenter	128 DCD 103 retrieved 74 transplanted (23 declined)	ECMO for PGD 309 ± 56	ECMO warm + cold ischemic time 48 ± 11	standard OCS perfusate approximately 1.2–1.5L of donor blood before the admin- istration of cardioplegia. tirofiban in addition to heparin to prevent leucocyte filter clotting 1L of cold crystalloid cardioplegia	Lactates trend and subjective evaluation of ventricular contractility	1- and 5-year survival of DCD HTx recipients was 94% and 88%, comparable to that of a contemporary cohort of DBD: 87% and 81% (p-value nom significant) The requirement for ECMO for severe primary graft dysfunction (sPGD) occurred in 12 of 74 (16%) of cases overall. ECMO requirement was significantly lower in the contemporary cohort (4/51, 8%) compared to the initial cohort (8/23, 35%) (P = 0.0064) Patients that required ECMO for sPGD also had a significantly higher cardiopulmonary bypass time compared to those who did not; 254 (228–301) versus 158 (132–183) min, respectively (P < 0.0001) incidence of PGD: 8% (4/51)	*Survival is comparable to that of traditional BD donors; asystolic warm ischemic time has an important role to play in initial organ dysfunction; tirofiban is a safe addition to the blood collection protocol and helps reduce filter clotting, and, poor lactate profiles on NMP may indi-* *cate an underlying pathology*	[13]
	297 DBD	no ECMO 276 ± 56	no ECMO warm + cold ischemic time 58 ± 13					
Case report	1 DCD, patient with a left ventricular assist device	Not specified	17h 03 min (total cross clamp)	Not specified	Lactates trend	global dysfunction of the heart was observed during early postoperative period, probably resulting from long perfusion of the donor heart, and the fact that the organ was harvested from a marginal donor. Therefore, a decision was made to initiate central ECMO. The recipient had two revisions due to bleeding, and was successfully weaned from ECMO on the third day Grade 1R rejection was observed according to endomyocardial biopsy, but the patient was hemodynamically stable, so he did not require additional treatment		[68]
Retrospective multicenter	A. 68 OCS	381 min	115 min pre e post OCS ischemia	standard OCS perfusate	Lactates trend	Similar 30 days survival (A: 92.4% vs. B: 90.2%; *p* = 0.745) postoperative MCS (%) (A: 25.0 vs. B: 39.2; *p* = 0.112) and postoperative dialysis (chronic) (%) (A: 4.4 vs. B: 27.5; *p* < 0.001) were numerically better inOCS, without any difference in the occurrence of early rejection (<1R A: 23.5; B: 25.5, p = 0.83)	*OCS heart allowed safe transplantation of surgically complex recipients with excellent 1-year outcomes, despite long preservation times and unfavourable donor characteristics*	[69]
	B. 51 Conventional		228 min ischemic non OCS					
Unblinded, randomized, controlled trial, multicenter	90 DCD 80 transplanted	Not specified	Not specified	standard OCS perfusate cold crystalloid del Nido cardioplegia solution (containing Plasma- Lyte A, mannitol, magnesium sulfate, sodium bicarbonate, potassium chloride, and lidocaine)	Lactates trend	Risk-adjusted 6-month survival in the as-treated population was 94% (95% confidence interval [CI], 88–99) among DCD, as compared with 90% (95% CI, 84–97) among DBD (least-squares mean difference, −3 percentage points; 90% CI, −10 to 3; P < 0.001 for noninferiority [margin, 20 percentage points]) No substantial between-group differences in the mean per-patient number of serious adverse events associated with the heart graft at 30 days after transplantation PGD moderate 6% DCD vs. 5% DBD; severe 15% DCD vs. 5% DBD.	*Risk-adjusted survival at 6 months after transplantation with a donor heart that had been reanimated and assessed with the use of extracorporeal nonischemic perfusion after circulatory death was not inferior to that after standard-care transplantation with a donor heart that had been preserved with the use of cold storage after brain death*	[70]
	90 DBD 86 transplanted							
Retrospective, single center	122 DCD 21 OCS 101 TA-NRP followed by CSS	Not specified	Not specified	Not specified	Not specified	No significant differences between groups in 1-year survival (94% DCD vs. 92% DBD, p = 0.50), incidence of severe PGD (6% DCD vs. 6%DBD, p = 0.99), treated rejection during the first year (13%DCD vs. 18% DBD, p = 0.21), or likelihood of cardiac allograft vasculopathy at 1 year after transplantation (15% DCD vs. 14% DBD, p = 0.96)	*In the largest single-center comparison of DCD and DBD HTx to date, outcomes among DCD recipients are noninferior to those of DBD recipients*	[71]
	263 DBD 10 OCS 253 CSS							
Retrospectivemulticenter	215 potential DCD hearts were offered of which 98 (46%) accepted 57 (27%) donor hearts retrieved and 50 (23%) DCD transplanted	258 (216–306)	warm ischemic time 28 (24–34) + cold ischemic time 13 (9–19)	standard OCS perfusate St Thomas’ cold crysralloid + erythropoietin + glyceryl trinitrate	Lactates trend	No difference in the 30-day survival rate between DCD and DBD (94% vs. 93%) or 90 days survival (90% vs. 90%) respectively 1 year survival 84% DCD vs. 84% DBD, p = 0.91 Higher rate of ECMO use post-DCD heart transplants compared to DBD (40% vs. 16%, p = 0.0006) Hemofiltration 60% DCD vs. 45% DBD, p = 0.08 Treated rejection episode in 30 days 8% DCD vs. 14% DBD, p = 0.41; in 90 days 9% DCD vs. 24% DBD, p = 0.06		[72]
	179 DBD							
Retrospective UNOS registry	558 procurements 65% DPP (363) 25% NRP (195)	Not specified	Not specified	Not specified	Lactates trend	Among 558 DCD procurements, recovery occurred in 89.6%, and 92.5% of recovered hearts were utilized for transplant NRP was also associated with higher odds for heart utilization after recovery compared with DPP (OR, 3.79; 95% CI, 1.40-10.24; P = 0.009)	*NRP procurements have a higher yield for DCD heart transplantation compared with direct procurement and perfusion*	[73]

BVAD, Biventricular Assiste Device; CAD, coronary artery disease; CPB, Cardio-Pulmonary Bypass; CSS, cold static storage; CRRT, continuous renal replacement therapy; DBD, donor after brain death; DCD, donor after circulatory death; ECMO, ExtraCorporeal Membrane Oxygenation; HTx, Heart transplant; IABP, Intra Aortic Balloon Pump; LVAD, Left Ventricular Assist Device; LV, left ventricle; MCS, Mechanical Circulatory Support; NA, not applicable; NRP, Normothermic Thoracoabdominal Regional Perfusion; OCS, Organ Care System; PGD, Primary graft dysfunction; RV, right ventricle; WIT, warm ischemic time. At the moment the only clinical approved MP, is OCS (Organ Care System).

Parallel to these studies, parameters were examined that characterize DCD grafts in comparison to DBD settings [57, 62, 64, 66, 67, 69–71] or between DCD grafts preserved via cold storage versus those preserved using MP [12, 60, 69, 71, 72]. Among the most relevant parameters, Dhital et al. demonstrated a differential role of lactates during OCS in DCD hearts, showing that higher lactate values (>5 mM) in DCD grafts were still acceptable as long as there was a downward trend in lactate levels and an arteriovenous differential favoring lactate metabolism as an energy source, rather than its accumulation, which would indicate myocardial injury [61]. Additionally, studies have been published examining diagnostic insights into DCD organs under MP, such as coronary angiography or cardiac magnetic resonance imaging [62, 65]. Both short- and long-term outcomes, including survival rates and rejection episodes, were found to be comparable to outcomes from DBD donors [13, 56–72].

## Discussion

In this review the role of EVHP for heart preservation in DCD clinical settings resulted in evaluation of grafts before HT, extended *ex-situ* preservation of the graft before implantation, and resuscitation of the graft. However, in DCD pre-clinical settings EVHP role becomes deeply investigate biological mechanisms causing IRI in order to test potential treatment to reduce or prevent it, improving cardiac recovery, and decreasing the risk of PGD or delayed graft failure.

### *Ex-Vivo* Heart Perfusion Technologies

In DCD the inevitable warm ischemia time (time between withdrawal of life support and circulatory arrest) makes the already energy-depleted heart poorly tolerant of additional cold ischemia caused by transport with well-established cold static storage (CSS) [10]. Alternative ways of organ preservation can be preferred in this context to improve patients’ outcomes. *Ex-situ* MP can be divided into normothermic MP (NMP; 34°C–37.5°C), hypothermic MP (HMP; 1–8°C) and subnormothermic MP (sNMP; 20°C–33.5°C) [74]. NMP is defined as heart preservation in a beating, unloaded, metabolically active state on a portable device. Currently, the only commercially available option for clinical practice is the Organ Care System (OCS; TransMedics, Andover, MA) [8, 70, 75]. The OCS device has recently been awarded approval by the Food and Drug Administration for use in DCD transplantation. HMP is defined as heart preservation in a non-beating state with low myocardial metabolism and perfused with oxygenated nutrient-rich perfusate. Current available devices are.

• The XVIVO NIHP system (XVIVO Perfusion AB, Göteborg, Sweden), which is still under clinical investigation (ongoing clinical trial),
• The Paragonix SherpaPerfusion Cardiac Transport System (Paragonix Technologies, Cambridge, MA), still not used in clinical practice with experience limited to swine models,
• The LifeCradle system (Organ Transport Systems, Inc, Frisco, TX), which has been studied in human hearts only *in vitro* settings [10].
  Subnormothermic MP at the moment is limited to other organs (liver, kidney).

### Pre-Transplant Heart Evaluation

One of the main issues in DCD is graft quality evaluation before implantation. During DCD, organs can either be directly procured (Direct procurement and perfusion, DPP) and Thoracoabdominal normothermic regional perfusion (TA-NRP) can be used. The latter consists in using veno-arterial extracorporeal membrane oxygenation (VA-ECMO) to re-perfuse the allograft in the donor immediately after pronouncement of circulatory death, with the goal of allowing the heart to recover from the warm ischemic injury, allowing for a detailed evaluation of cardiac function and pump function *in situ*. Then, the graft is procured if deemed suitable for use [76] and either maintained with *ex situ* MP or with CSS during transport.

If DPP is chosen, the pre-retrieval function is incompletely assessed. In this setting, NMP enables a quantitative heart assessment via measurement of heart rate and rhythm, pump flow, coronary flow rate, aortic pressure, hematocrit, temperature oxygen saturation and arteriovenous lactate levels trend. Nevertheless, ventricular contractility is evaluated only via subjective visual examination – epicardial echocardiography is technically demanding and not standardized – in the setting of an unloaded heart (as the left ventricle is actively vented during perfusion in resting mode) [10, 17, 75]. A recent systematic review highlighted the role of coronary angiography performed while the graft was already connected to NMP [77]. So far, nine total cases of ex‐situ coronary angiography of donor human hearts plus one experimental porcine model have been reported. This technique is particularly useful in DCD because coronary angiography is not always available due to ethical issues and availability in procurement centers [15, 57]. In some institutions even echocardiogram is not routinely available because it is considered an unacceptable antemortem intervention [59].

### Pre-Clinical Studies

Although CSS is universally accepted as a safe and effective method of heart preservation, PGD continues to affect approximately 3% of clinical heart transplants performed worldwide, and accounts for 26% of deaths in the first 30 days after transplantation [78]. It has been demonstrated that a continuous perfusion with oxygen and metabolites is a physiological support of aerobic metabolism needed for the maintenance of cell integrity and vital cell functions during the transport period [79–83]. Other potential advantages are myocardial cooling through the native coronary circulation and the ongoing washout of metabolic byproducts resulting in improvement of myocardial preservation and microvascular protection. In CSS settings there is a change in myocardial metabolism and viability: ATP levels are depleted and fail to recover despite reperfusion with all necessary substrates for energy repletion, markers of oxidative injury (MDA), apoptosis (caspase-3), and endothelial dysfunction (ET-1) are induced and are further exacerbated by reperfusion [23, 51, 84]. In contrast, EVHP preserves ATP and maintains baseline tissue pH. As a result, these hearts show a rapid restoration of ATP and shorter period of acidosis during reperfusion, suggesting decreased oxygen debt and improved microvascular recovery with a strong correlation with systolic function [85]. Although EVHP is demonstrated to preserve systolic function, a consistent concern is represented by edema, which may negatively affect post-transplant diastolic recovery [21, 29, 86]. In addition, MP does not evaluate diastolic function.

### Pre-Transplant Heart Therapies

NMP has also been considered in experimental settings to prevent IRI in DCD and subsequently leads to a lower incidence of PGD and delayed graft failure. This has been achieved in pre-clinical scenarios by enriching the MP perfusate with specific molecules interfering with core steps of IRI, as the nucleotide-binding oligomerization domain-like receptor pyrin domain-containing 3 (NLRP3) inflammasome [55]. Oxidative stress can also be targeted by adding melatonin to cardioplegia and perfusate during EVHP [42]. Even cardiac gene therapy is emerging as a promising approach. It can be obtained both with addition of siRNA in cardiac NMP or via adeno-associated virus specific gene transfer to myocytes [87]. Target genes are involved in inflammatory and apoptotic signal proteins (such as C3, nuclear factor κB-p56, caspase-8, and caspase-3). Additionally, a limited number of studies have reported on the use of mesenchymal stem cells during EVHP due to their anti-inflammatory and immunomodulatory effects both by cell-to-cell contact and by secreting substances [88]. At the moment the focus of these studies is still on feasibility and safety aspects.

To date, more than 50 DCD heart transplants have been performed in Australia and United Kingdom by means of NMP technology with excellent short-term and long-term (4 years) outcomes, comparable with those of DBD transplantation [12, 13, 59].

However, given the high cost of the MP devices themself, comparative cost analyses are needed to elucidate their cost effectiveness. The OCS-DCD US trial was the first randomized trial comparing DCD heart transplant to DBD standard criteria heart transplant clinical outcomes [75]: to date, 180 patients (90 DCD vs. 90 DBD) were enrolled and transplanted. One year patient and graft survival were greater than 90%, with a higher rate compared with DBD. Unfortunately, incidence of moderate-to-severe ISHLT PGD was around 20% in DCD versus 9.1% in DBD, raising the idea that warm ischemia (even though <30 min) can negatively affect early graft function. Chew et al. observed that in the recovery of hearts for DCD, WIT was a crucial determinant of outcome [58]. The need for post-transplant mechanical circulatory support rises when the asystole to cardioplegia (AP) time exceeds 15 min. The interval between circulatory arrest and AP time became a determinant of delayed graft function and the need for short-term ECMO. The full recovery of DCD hearts after short-term support using ECMO was suggestive of delayed graft function as opposed to PGD. On the other hand, Coniglio et al. showed that there were no differences in cardiac MRI findings realized <60 days from HT between those who underwent DCD transplantation using OCS device, DBD using OCS device and DBD transported via CSS, including presence of gadolinium hyperenhancement after transplant (all p > 0.050) [64].

## Conclusion

To conclude, EVHP in the setting of DCD heart transplantation model can be a valid tool for organ preservation and transport. The role of pre-clinical research will be crucial to reduce IRI, achieve organ reconditioning and reduce incidence of PGD and delayed graft function.

## Abbreviations

CSS, Cold Static Storage; DBD, Donation after brain death; DCD, Donation after circulatory death; ESHF, end-stage heart failure; EVHP, *ex-vivo* heart preservation; HMP, hypothermic MP; IRI, ischemia-reperfusion injury; MP, Machine Perfusion; NMP, normothermic MP; NRP, normothermic regional perfusion; PGD, primary graft dysfunction; VA-ECMO, veno-arterial extracorporeal membrane oxygenation.
