# Supplementary material for: Ex-Vivo Heart Perfusion Machines in DCD Heart Transplantation Model: The State of Art
Source: Transpl Int. 2025 Aug 13;38:12987. doi: 10.3389/ti.2025.12987 (PMC12382452; doi:10.3389/ti.2025.12987)
Supplement: Supplementary file 1 [file Table1.pdf]

Supplementary table – Comparison of perfusion strategies and clinical outcomes in pre-clinical studies using MP in DCD.

ATP – adenosine triphosphate; **BCD – beating-heart cardiac donor;** CBD – control beating-heart donor; **CPB – Cardiopulmonary bypass;** CSS – cold static storage; **CVR – Coronary vascular resistance;** DBD – donor after brain death; **DCD – donor after circulatory death;** DP – developed pressure; **DPP – direct procurement;** HR – heart rate; **HTK – Histidine-tryptophan-ketogluterate**; LDH – lactate and lactate dehydrogenase**; LV - left ventricle;** LVDP – left ventricular diastolic pressure; **MP – Machine Perfusion;** MVO_2 – Myocardial oxygen consumption; NA – not applicable; NR – Not Reported; **NRP – Normothermic Regional Perfusion;** OCS – Organ Care System (OCS); **PGD – Primary graft dysfunction;** RV – right ventricle; **ST – Saint Thomas;** UW – University of Wisconsin solution; **WIT – warm ischemic time.**

Animal Model	Machine perfusion	Study Design	Study Objective	Perfusate used<br><br>Cardioplegia used	Ischemic Time<br>Perfusion Time	Myocardial functional assessment	Reference
Wistar Rat	Custom made MP	Ex vivo WIT followed by MP (n=NR)	Primarily graft evaluation	P-free Krebs-Henseleit solution<br><br>NA	5-43 min<br><br>40 min	Power output	Houston RJ, Skotnicki SH, Heerschap A, Oeseburg B. Coronary flow response after myocardial ischemia may predict level of functional recovery. Adv Exp Med Biol. (1997);411:121–127.
Domestic pig	Custom made MP	Parallel, 2-arm study<br>1. Normal (non-DCD, non-DBD) hearts (n=9)<br>2. DCD hearts (n=37)	Primarily graft evaluation	STEEN Solution<br><br>Crystalloid cardioplegic solution	1. 4.6±0.2 min<br>2. 27.6±0.3 min	Systolic function, myocardial work, diastolic function, MVO2, CVR	White CW, Ambrose E, Müller A, et al. Assessment of donor heart viability during ex vivo heart perfusion. Can J Physiol Pharmacol. (2015);93:893–901.
Human	Custom made MP	DCD Heart non transplanted (n=1)	Primarily graft evaluation	Oxygenated organ perfusion solution<br><br>Custom-designed cardioplegic solution	17 min<br><br>150 min	Perfusate lactate levels<br>Isovolumic developed pressure	Rosenfeldt F, Ou R, Woodard J, Esmore D, Marasco S. Twelve-hour reanimation of a human heart following donation after circulatory death. Heart Lung Circ (2014);23:88–90.
Human	Custom made MP	1. DBD (n = 5)<br>2. DCD donor hearts (n = 5)	Primarily graft evaluation	Human red blood cells and crystalloid<br><br>UW solution	1. WIT 34 ± 3 min<br>2. cold ischemic time 211±31 min.<br><br>120 min	Left ventricular function	Osaki S, Locher MR, Lushaj EB, Akhter SA, Kohmoto T. Functional evaluation of human donation after cardiac death donor hearts using a continuous isolated myocardial perfusion technique: Potential for expansion of the cardiac donor population. The Journal of Thoracic and Cardiovascular Surgery (2014);148:1123–30.
Lewis rat	Custom made MP	Parallel, 3-arm study:<br>1. 4 h CSS without WIT<br>1. 4 h CSS (n=11)<br>2. 1 h normothermic reperfusion and 4 h CSS (n=11)	Primary graft evaluation	Autologous blood with Krebs–Henseleit buffer<br><br>Custodiol	25 min<br><br>60 min	Troponin T, LDH, creatine kinase, and K+, oxidative stress tissue energy state, histology	Tolboom H, Makhro A, Rosser BA, et al. Recovery of donor hearts after circulatory death with normothermic extracorporeal machine perfusion. Eur J Cardiothorac Surg. (2015);47:173–179.
Pig	OCS	DCD (n=5)	Primarily graft evaluation	Warm oxygenated blood<br><br>In-house cardioplegia	25±7 min<br><br>240 min	Visual contractility, metabolic profiles	García Sáez D, Elbetanony A, Lezberg P, et al. Ex vivo heart perfusion after cardiocirculatory death; a porcine model. J Surg Res (2015);195:311–4.
Landrace pig	HeartPort© System	Parallel, 2-arm study<br>1. 4 h cold CSS (n=8)<br>2. 4 h cold MP (n=8)	Primarily graft evaluation	1 l asanguineous KPS-1©.<br><br>1-l HTK solution	1.14.6 ± 4.5 min<br>2. 21.9 ± 11 min<br><br>1. NA<br>2. 240 min	Myocardial oedema; myocardial tissue lactate, creatine and phosphocreatine, tissue high-energy phosphate content, ventricular function	Van Caenegem O, Beauloye C, Bertrand L, et al. Hypothermic continuous machine perfusion enables preservation of energy charge and functional recovery of heart grafts in an ex vivo model of donation following circulatory death. Eur J Cardiothorac Surg. (2016);49:1348–1353.
Sprague-Dawley Rat	Custom made MP	Randomized, prospective, parallel studies (n=75):<br>1. 5 min WIT (n=15)<br>2. 10 min WIT (n=15)<br>3. 20 min WIT (n=15)<br>4. Beating Heart Control (n=15)<br>5. Positive ischemic control (n=15)	Primarily graft evaluation	Krebs-Henseleit buffer.<br><br>Celsior	According to protocol	Left ventricular function assessment; ultra-Sensitive Cardiac Troponin-I Assay, Histologic Analysis	Kearns MJ, Miller SD, Cheung A, et al. A rodent model of cardiac donation after circulatory death and novel biomarkers of cardiac viability during ex vivo heart perfusion. Transplantation. (2017);101:e231–e239.

Animal Model	Machine perfusion	Study Design	Study Objective	Perfusate used<br><br>Cardioplegia used	Ischemic Time<br>Perfusion Time	Myocardial functional assessment	Reference
Pig	Custom made MP	n = 8	Primarily graft evaluation	Hypothermic, oxygenated Custodiol-N<br><br>Custodiol	30 min<br><br>240 min	LV contractility, lactate levels, mLDP	Saemann L, Kohl M, Veres G, et al. Prediction Model for Contractile Function of Circulatory Death Donor Hearts Based on Microvascular Flow Shifts During Ex Situ Hypothermic Cardioplegic Machine Perfusion. J Am Heart Assoc (2022);11:e027146.
Sprague–Dawley Rat	Custom made MP	1. controlled DBD (n=36)<br>2. DCD(n=36)<br><br>Perfusion solutions:<br>- n=6 ST, 15°C<br>- n=6 ST, 4°C<br>- n=6 UW, 15°C<br>- n=6 UW, 4°C,<br>- n=6 polyethylene glycol-20k (PEG), 15°C,<br>- n=6 PEG, 4°C	Primarily graft evaluation	ST, UW, and PEG<br><br>NA	1. NA<br>2. 25 min<br><br>60 min	Heart weight, regional tissue flow temperature, flow rate, and perfusion pressures, cardiac troponin I, histology	Cholyway R, Akande O, Mauro AG, et al. Assessment of machine perfusion conditions for the donation after circulatory death heart preservation. Artificial Organs (2022);46:1346–57.
Porcine	Custom made MP	1. normothermic blood (DCD-B) (n=5);<br>2. hypothermic Custodiol (n=5),<br>3. Novel Custodiol (n=5).	Primarily graft evaluation	Different in each group<br><br>HTK solution	30 min<br><br>240 min	microvascular and contractile evaluation, Immunohistochemical Staining Gene Expression Analysis	Saemann L, Georgevici A-I, Hoorn F, et al. Improving Diastolic and Microvascular Function in Heart Transplantation with Donation after Circulatory Death. IJMS (2023);24:11562.
Large White piglets	OCS	1. warm ischemia DCD group (n = 6);<br>2. brainstem death DBD group (n = 6);<br>3.Control group, (n = 6).	Primarily graft evaluation	Enriched saline solution + Blood<br><br>Modified Del Nido cold cardioplegia solution	23 minutes in DCD<br><br>240 min	Metabolic profile in plasma and heart bioptic samples	Hautbergue T, Laverdure F, Van SD, et al. Metabolomic profiling of cardiac allografts after controlled circulatory death. The Journal of Heart and Lung Transplantation (2023);42:870–9.
Abattoir pig	Custom made MP	1. Immediate blood reperfusion with hemodiluted homologous whole pig blood (n = 7).<br>2. Additional perfusion with UW solution, followed by blood reperfusion (n = 6).<br>3. Additional CSS, before blood reperfusion (n = 6).	Primarily protocol optimization	UW solution.<br>Homologous whole pig blood (1500 mL) + K-free Krebs–Henseleit solution	25 min<br><br>240 min	LVDP, contractility, coronary flow, histology	Scheule AM, Haas J, Zurakowski D, et al. A non-heart-beating donor model to evaluate functional and morphologic outcomes in resuscitated pig hearts. J Invest Surg (2002);15:125–35.
Wistar Rat	Custom made MP	Isolated hearts (n=31) No-flow ischemia for different minutes	Primarily protocol optimization	KH buffer supplemented<br><br>NA	30, 50, 55, 60 min<br><br>60 min	LV pressure curves and HR	Dornbierer M, Stadelmann M, Sourdon J, et al. Early reperfusion hemodynamics predict recovery in rat hearts: a potential approach towards evaluating cardiac grafts from non-heart-beating donors. PLoS One. (2012);7:e43642.
Domestic Pig	Custom made MP	1. ST Hospital solution No. 2, cold hyperkalemic cardioplegic arrest (n = 9).<br>2. Adenosine-lidocaine cardioplegia, continuous myocardial perfusion (n = 8).	Primarily protocol optimization	STEEN solution autologous blood<br><br>1. hyperkalemic cardioplegia<br>2. adenosine-lidocaine cardioplegia	15 min<br><br>120 min	Myocardial oedema, Troponin I, oxidative stress, histologic analysis, assessment of ventricular Function Ventricular	White CW, Ali A, Hasanally D, et al. A cardioprotective preservation strategy employing ex vivo heart perfusion facilitates successful transplant of donor hearts after cardiocirculatory death. J Heart Lung Transplant (2013);32:734–43.
Yorkshire Landrace pig	Custom made MP	1. DCD hearts in CSS (n=11)<br>2. DCD hearts in MP (n=6)<br>3. DCD hearts in retrograde oxygen persufflation (n=6).	Primarily protocol optimization	1. NA<br>2. UW solution<br><br>AQIX RS-I solution.	1. 10 min ± 0<br>2. 14 min ± 4<br>3. 22 min ± 4<br><br>120 min	Heart reanimation	Mownah OA, Khurram MA, Ray C, et al. Development of an ex vivo technique to achieve reanimation of hearts sourced from a porcine donation after circulatory death model. J Surg Res (2014);189:326–34.

Animal Model	Machine perfusion	Study Design	Study Objective	Perfusate used<br><br>Cardioplegia used	Ischemic Time Perfusion Time	Myocardial functional assessment	Reference
Juvenile Landrace pig	OCS	DCD heart<br>1. normothermic EVHP (n = 8)<br>2. CSS (n = 3)	Primarily protocol optimization	TransMedic priming and maintenance solutions<br><br>Celsior	30 min<br><br>240 min	Metabolic profiles; ability to wean off CPB post-transplantation, and to maintain hemodynamic stability for 3 h post-weaning; heart contractility,cardiac output	Iyer A, Gao L, Doyle A, et al. Normothermic Ex Vivo Perfusion Provides Superior Organ Preservation and Enables Viability Assessment of Hearts From DCD Donors. American Journal of Transplantation (2015);15:371–80.
Lewis Rat	Custom made MP	1. CSS (n=14)<br>2. reperfusiona and CCS (n=14)<br>7. ischemic hearts and CSS (controls)	Primarily protocol optimization	1. NA<br>2-6. autologous blood with Krebs–Henseleit buffer<br><br>Custodiol	1-6: 25 min<br>7: NA<br><br>60 min	Myocardial injury, oxidative stress, tissue energy state, tissue oedema.	Tolboom H, Olejníčková V, Reser D, et al. Moderate hypothermia during ex vivo machine perfusion promotes recovery of hearts donated after cardiocirculatory death. Eur J Cardiothorac Surg.(2016);49:25–31.
Pig	Custom made MP	DCD hearts:<br>1. 5°C (n = 6),<br>2. 25°C (n = 5),<br>3. 35°C (n= 7)	Primarily protocol optimization	STEEN solution and whole donor blood<br><br>Normokalemic adenosine–lidocaine cardioplegia	1. 5°C = 28<br>2. 25°C = 29<br>3. 35°C = 28<br><br>360 min	Myocardial oedema, troponin I, electron microscopy, CVR, myocardial metabolism, myocardial function.	White CW, Avery E, Müller A, et al. Avoidance of Profound Hypothermia During Initial Reperfusion Improves the Functional Recovery of Hearts Donated After Circulatory Death. American Journal of Transplantation (2016);16:773–82.
Yorkshire pig (pediatric)	Custom made MP	n = 12<br>1. pressure-targeted perfusion 40 mm Hg<br>2. flow-targeted perfusion 10 ml/kg/min	Primarily protocol optimization	Whole blood<br>Cardioplegic solution of the Hospital for Sick Children.	1. 22.8 ± 1.3<br>2. 25.7 ± 3.4<br><br>1. 137.2 ±7.8<br>2. 130.3 ±8.7	Systolic and diastolic myocardial performance, heart weight, blood gas analysis and lactate concentration, Troponin-I, Histology.	Kobayashi J, Luo S, Akazawa Y, et al. Flow-targeted pediatric ex vivo heart perfusion in donation after circulatory death: A porcine model. The Journal of Heart and Lung Transplantation (2020);39:267–77.
Yorkshire pig	CBP with resting and working mode	n = 6<br>- blood cardioplegic solution<br>- leukocyte-depleted hyperxemic oxygenated blood (<br>- perfusion pressure 60 mm Hg, temperature 37°C<br>- controlled reperfusion, switched into working mode	Primarily protocol optimization	Leukocyte-depleted hyperxemic oxygenated blood<br><br>Modified ST Hospital solution	22 ± 1 min<br><br>70 min + 60 min	Cardiac function, lactate level, and myocardial edema, systemic and pulmonary vascular resistance	Kobayashi Y, Kotani Y, Sakoda N, et al. Ex vivo evaluation of the biventricular cardiac function for donation after circulatory death model: An experimental study. Artif Organs (2021);45:373–81.
Pig	Custom made MP	EVHP-system	Primarily protocol optimization	Blood<br><br>Custodiol	30 min<br><br>240 min	Left ventricular contractile function, myocardial microcirculation	Saemann L, Wenzel F, Kohl M, et al. Monitoring of perfusion quality and prediction of donor heart function during ex-vivo machine perfusion by myocardial microcirculation versus surrogate parameters. The Journal of Heart and Lung Transplantation (2021);40:387–91.
Human	OCS	A. DBD (n=6)<br><br>B. DCD (n=1)	Primarily protocol optimization	OCS Priming:<br>A. Leucocyte filtered Donor Blood + priming solution<br><br>B. Packed red blood cells	A. 270 min (IQR 216-343)<br>B. 270 min	Differential venous−arterial lactate profile, ex-vivo coronary angiography	Dang Van S, Gaillard M, Laverdure F, et al. Ex vivo perfusion of the donor heart: Preliminary experience in high-risk transplantations. Archives of Cardiovascular Diseases (2021);114:715–26.
Wistar Rat	Custom made MP	n=50<br>1. CSS,<br>2. HOPE<br>3. HNPE<br>4. HOPE + AA5	Primarily protocol optimization	S. Thomas No 2 solution Perfusate of HOPE<br><br>S.Thomas No 2 cardioplegic solution	21 min<br><br>1. NA<br>2.-4.60 min	Cardiac function, coronary flow, cardiac oxygen consumption, tissue edema, circulating markers of cellular and mitochondrial injury and oxidative stress, measurement of tissue succinate, ATP and ADP levels, and mitochondrial ROS emission.	Wyss RK, Méndez Carmona N, Arnold M, et al. Hypothermic, oxygenated perfusion (HOPE) provides cardioprotection via succinate oxidation prior to normothermic perfusion in a rat model of donation after circulatory death (DCD). American Journal of Transplantation (2021);21:1003–11.
Human	Heart Assist	DCD hearts (n=2)	Primarily protocol optimization	Perfadex Plus<br><br>Custodiol HTK solution	1. 27 min<br>2. NA<br><br>1. 408 min<br>2. 432 min	Conic balloon inserted in the LV through the mitral valve annulus for functional evaluation	van Suylen V, Bunnik EM, Hagenaars JAM, et al. Ex Situ Perfusion of Hearts Donated After Euthanasia: A Promising Contribution to Heart Transplantation. Transplant Direct (2021);7:e676.

Animal Model	Machine perfusion	Study Design	Study Objective	Perfusate used<br><br>Cardioplegia used	Ischemic Time Perfusion Time	Myocardial functional assessment	Reference
Wistar rat	Custom made MP	1. no-warm ischemia<br>2. ischemia group + St.Thomas solution<br>3. ischemia +A-L solution at +4°C<br>4. ischemia + A-L solution at +22°C	Primarily protocol optimization	Modified Krebs-Henseleit buffer<br><br>1. no-warm ischemia<br>2. St. Thomas solution +<br>3. A-L solution<br>4. A-L solution	1. 0 min<br>2.3.4. 18 min<br><br>1.2. 60 min<br>3.4. 30 min	Endothelial-related parameters	Méndez-Carmona N, Wyss RK, Arnold M, et al. Effects of graft preservation conditions on coronary endothelium and cardiac functional recovery in a rat model of donation after circulatory death. J Heart Lung Transplant (2021);40:1396-1407.
Sprague Dawley rats	Custom made MP	Case groups (AMO-treated): CBD (n=NA), DCD (n=NA)<br>Control groups: BCD (n=NA), DCD (n=NA)	Primary protocol optimization	Modified Krebs-Henseleit solution +/- AMO	25 min<br><br>1. 60 min<br>2. 90 min	LV function, mitochondrial oxidative phosphorylation, CRC assay, SSM, IFM.<br>Infarct size measurement	Akande O, Chen Q, Cholyway R, et al. Modulation of mitochondrial respiration during early reperfusion reduce injury in donation after circulatory death hearts. J Cardiovasc Pharmacol (2022);80:148-157.
Danish Landrace pig	XVIVO Heart preservation system	1. NRP followed by CSS (n=9)<br>2- NRP followed by HMP (n=7)<br>3. DPP followed by HMP (n=8)	Primarily protocol optimization	1. NA<br>2. NA<br>3. XVIVO Heart Solution<br><br>1. HTK solution	1. 13 (12-4)<br>2. 18 (17-19)<br>3. 19 (17-20)<br><br>1. NA<br>2. 180 min<br>3. 180 min	Cardiac function, biochemical analysis	Moeslund N, Ertugrul IA, Hu MA, et al. Ex-situ oxygenated hypothermic machine perfusion in donation after circulatory death heart transplantation following either direct procurement or in-situ normothermic regional perfusion. The Journal of Heart and Lung Transplantation (2023);42:730–40.
Landrace pig	HeartPort System©	1. 4 °C at 10–15 mmHg, deep hypothermia (n =5);<br>2. 18 °C at 20–30 mmHg, moderate hypothermia (n=5);<br>3. 25 °C at 30–50 mmHg, mild hypothermia (n=5);<br>4. 35 °C at 50–70 mmHg, tepid temperature (n=5)	Primarily protocol optimization	KPS-1©<br><br>Custodiol	1. 27.8 ± 4.2<br>2. 26.6 ± 2.5<br>3. 26.2 ± 2.6<br>4. 26.0 ± 4.6<br><br>180 min	Cardiac metabolism,myocardial oedema	Mastrobuoni S, Johanns M, Vergauwen M, et al. Comparison of Different Ex-Vivo Preservation Strategies on Cardiac Metabolism in an Animal Model of Donation after Circulatory Death. J Clin Med (2023);12:3569.
Sprague-Dawley rats	Custom made MP	Parallel, 2-arm study<br>1. CBD n=10, DCD n=20 at 15, 20, 25, 30 and 35 min.<br>2. CBD n=8, DCD n=27<br>3. CBD n=5, DCD n=15	Primarily protocol optimization	Krebs–Henseleit solution	25 min<br><br>90 minutes (*60 min for mitochondrial assessment)	1. Infarct size<br>2. Mitochondrial function<br>3. Graft function assessment	Quader M, Akande O, Cholyway R, et al. Infarct size with incremental global myocardial ischemia times: cyclosporine A in donation after circulatory death rat hearts. Transplant Proc 2023;55:1495-1503.
Juvenile Yorkshire pig	OCS	Parallel, 2-arm study<br>1. DCD hearts reperfused (n=7)<br>2. DCD hearts reperfused with 5-nmol exenatide (n=7)	Primarily protocol optimization	Perfusate composition<br><br>Enriched cardioplegia solution	15 min<br><br>90 min	Left ventricular fraction,<br>Metabolic parameters,<br>Western blot,<br>Histopathology	Kadowaki S, Siraj MA, Chen W, et al. Cardioprotective actions of a glucagon-like peptide-1 receptor agonist on hearts donated after circulatory death. J Am Heart Assoc (2023);12:e027163.
Pig	Custom made MP	1. Normothermic EVHP with steroids (n=5)<br>2. Normothermic EVHP without steroids (n=8)	Primary therapeutic approach for re-conditioning	STEEN<br><br>MPS2	15 min<br><br>360 min	Myocardial oedema, perfusate cytokines, perfusate Troponin I, coronary blood flow, myocardial oxygen consumption and lactate metabolism, myocardial function, rates of pressure change in the LV, histopathologic analysis	Sandha JK, White CW, Müller A, et al. Steroids Limit Myocardial Edema During Ex Vivo Perfusion of Hearts Donated After Circulatory Death. Ann Thorac Surg (2018);105:1763–70.
Yorkshire Pig	Custom made MP	1. Postconditioning with Intralipid (n =5)<br>2. No treatment (n = 5).	Primary therapeutic approach for re-conditioning	Blood +Krebs-Henseleit solution<br><br>Normokaliemic crystalloid adenosine-lidocaine cardioplegia.	NR<br><br>30 min + 30 min	Cardiac biopsy, immunoblotting, histochemistry	Lucchinetti E, Lou P-H, Hatami S, et al. Enhanced myocardial protection in cardiac donation after circulatory death using Intralipid® postconditioning in a porcine model. Can J Anesth/J Can Anesth (2019);66:672–85.

Animal Model	Machine perfusion	Study Design	Study Objective	Perfusate used<br>Cardioplegia used	Ischemic Time Perfusion Time	Myocardial functional assessment	Reference
Yorkshire pigs	Custom made MP	1. vehicle alone (n = 8)<br>2. vehicle containing mitochondria (n=8).<br>3. serial injection of mitochondria (n = 6)<br>4. Sham group (n = 6).	Primary therapeutic approach for re-conditioning	Plasma-Lyte A 7.4<br>Del Nido cardioplegia	1-3. 20 min<br>4. NA<br>1-3. 240 min<br>4. NA	Myocardial functional parameters, perfusate serum lactates and myocardial oxygen consumption, histology, infarct size, metabolomic analysis.	Guariento A, Doulamis IP, Duignan T, et al. Mitochondrial transplantation for myocardial protection in ex-situ–perfused hearts donated after circulatory death. The Journal of Heart and Lung Transplantation (2020);39:1279–88.
Sprague-Dawley Rat	Custom made MP	1. Control group:<br>non-DCD hearts (n=8);<br>2. DCD-vehicle group (n=8);<br>3. DCD-melatonin group (n=8).	Primary therapeutic approach for re-conditioning	Blood from the donor modified Krebs–Henseleit solution<br>Custodiol	1. NA<br>2-3. 25 min<br>105 min	Developed pressure, HR, LV pressure, western blotting histologic and immunohistochemical analysis, cytokine expression	Lu J, Xu L, Zeng Z, et al. Normothermic ex vivo Heart Perfusion Combined With Melatonin Enhances Myocardial Protection in Rat Donation After Circulatory Death Hearts via Inhibiting NLRP3 Inflammasome-Mediated Pyroptosis. Front Cell Dev Biol (2021);9:733183.
Pig	Custom made MP	1. DCD with cytokine adsorption (CytoSorb®) (n = 5)<br>2. DCD without cytokine adsorption(n = 5)<br>3.-4. two control groups DCD hearts and native control hearts (n=5)	Primary therapeutic approach for re-conditioning	Normothermic blood perfusion<br>Custodiol	NR<br>240 min	Microvascular autoregulation immunoreactivity of arteriolar oxidative stress markers, concentration of cytokines in the perfusate, expression of genes.	Saemann L, Hoorn F, Georgevici A-I, Pohl S, Korkmaz-Icöz S, Veres G, et al. Cytokine Adsorber Use during DCD Heart Perfusion Counteracts Coronary Microvascular Dysfunction. Antioxidants (Basel) (2022);11:2280.
Lewis rat	Custom made MP	1. vehicle (dimethyl sulfoxide) (n= 5)<br>2. HSP90i (n= 6),<br>3. DCD-vehicle (n = 7)<br>4. DCD-HSP90i (n=9).	Primary therapeutic approach for re-conditioning	Ice-cold Krebs solution<br>Plasma-Lyte A	15 min<br>60 min	Western blot, mRNA expression of selected genes, myocardial contractility	Aceros H, Der Sarkissian S, Borie M, et al. Novel heat shock protein 90 inhibitor improves cardiac recovery in a rodent model of donation after circulatory death. J Thorac Cardiovasc Surg (2022);163:e187–97.
Naïve male Sprague Dawley rat	Custom made MP	1. IL-11 group (n = 15)<br>2. control group (n = 15)	Primary therapeutic approach for re-conditioning	normothermic K–H buffer solution<br>NA	25 min<br>60 min	Cardiac function, biochemical analysis, histopathologic analysis	Sakata T, Kohno H, Inui T, et al. Cardioprotective effect of Interleukin-11 against warm ischemia-reperfusion injury in a rat heart donor model. European Journal of Pharmacology (2023);961:176145.
Lewis Rat	Custom made MP	1. Control group: non-DCD hearts (n=6).<br>2. Vehicle group: saline + blood-based perfusate before EVHP (n=11)<br>3. MP-mcc950 group (n=10)<br>4. MP + PO (Post-operation)-mcc950 group (n=10)	Primary therapeutic approach for re-conditioning	Blood from the rat + modified Krebs–Henseleit solution<br>Custodiol	2-4. 15 min<br>1-4. 90 min	Oxidative stress, inflammatory response, apoptosis, immunofluorescence, western blot	Xu L, Zeng Z, Niu C, et al. Normothermic ex vivo heart perfusion with NLRP3 inflammasome inhibitor Mcc950 treatment improves cardiac function of circulatory death hearts after transplantation. Front Cardiovasc Med 2023;10:1126391.
